# Supplementary material for: Cu(II)-Loaded Polydopamine-Coated Urchin-like Titanate Microspheres as a High-Performance IMAC Adsorbent for Hemoglobin Separation
Source: Molecules. 2024 Apr 7;29(7):1656. doi: 10.3390/molecules29071656 (PMC11013688; doi:10.3390/molecules29071656)
Supplement: Supplementary file 1 [file molecules-29-01656-s001.zip › molecules-2923649-SI.pdf]

Supporting information

# Cu(II)-Loaded Polydopamine-Coated Urchin-Like Titanate Microspheres as a High-Performance IMAC Adsorbent for Hemoglobin Separation

*Qian Zhang [1, 2,†], Linlin Hu [1,2,†], Jianyu Yang [3], Pengfei Guo [1,2,*], Jinhong Wang [1,*]*

*and Weifen Zhang [1,2]*

[a] School of Pharmacy, Shandong Second Medical University, Weifang, 261053, China;

zhang_qian0424@163.com (Q.Z.); hull@sdsmu.edu.cn (L.H.);

zhangwf@sdsmu.edu.cn (W.Z.)

[b] Shandong Engineering Research Center for Smart Materials and Regenerative

Medicine, Weifang, 261053, China

[c] School of Materials Science and Engineering, Suzhou University of Science and

Technology, Suzhou, 215009, China; jianyuyang@usts.edu.cn

Correspondence: guopf@sdsmu.edu.cn (P.G.); wfmcwjh@sdsmu.edu.cn (J.W.)

† These authors contributed equally to this work.

Figure S1. TEM element mapping of Cu-PDA-UTMS.

Figure S2. SEM images of Na-TNT (a, b), Na-UTMS (c, d) and H-UTMS (e, f).

Figure S3. Photographs of H-UTMS (a), PDA-UTMS(b) and Cu-PDA-UTMS(c).

Figure S4. The circular dichroism (CD) of standard hemoglobin and hemoglobin solution after adsorption by the Cu-PDA-UTMS and recovery in 0.1% CTAB solution (The final hemoglobin solution is required to remove the CTAB molecule through dialysis).

Table S1. Elements content of H-UTMS, PDA-UTMS and Cu-PDA-UTMS by SEM/EDS

.

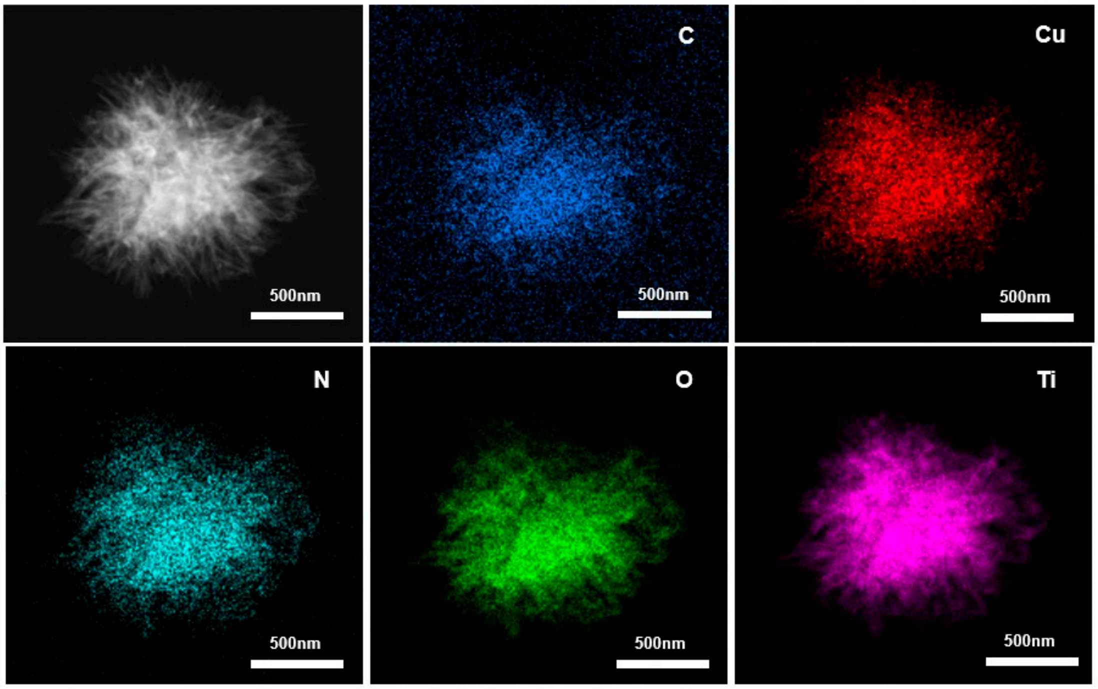


**Figure S1.** TEM element mapping of Cu-PDA-UTMS.

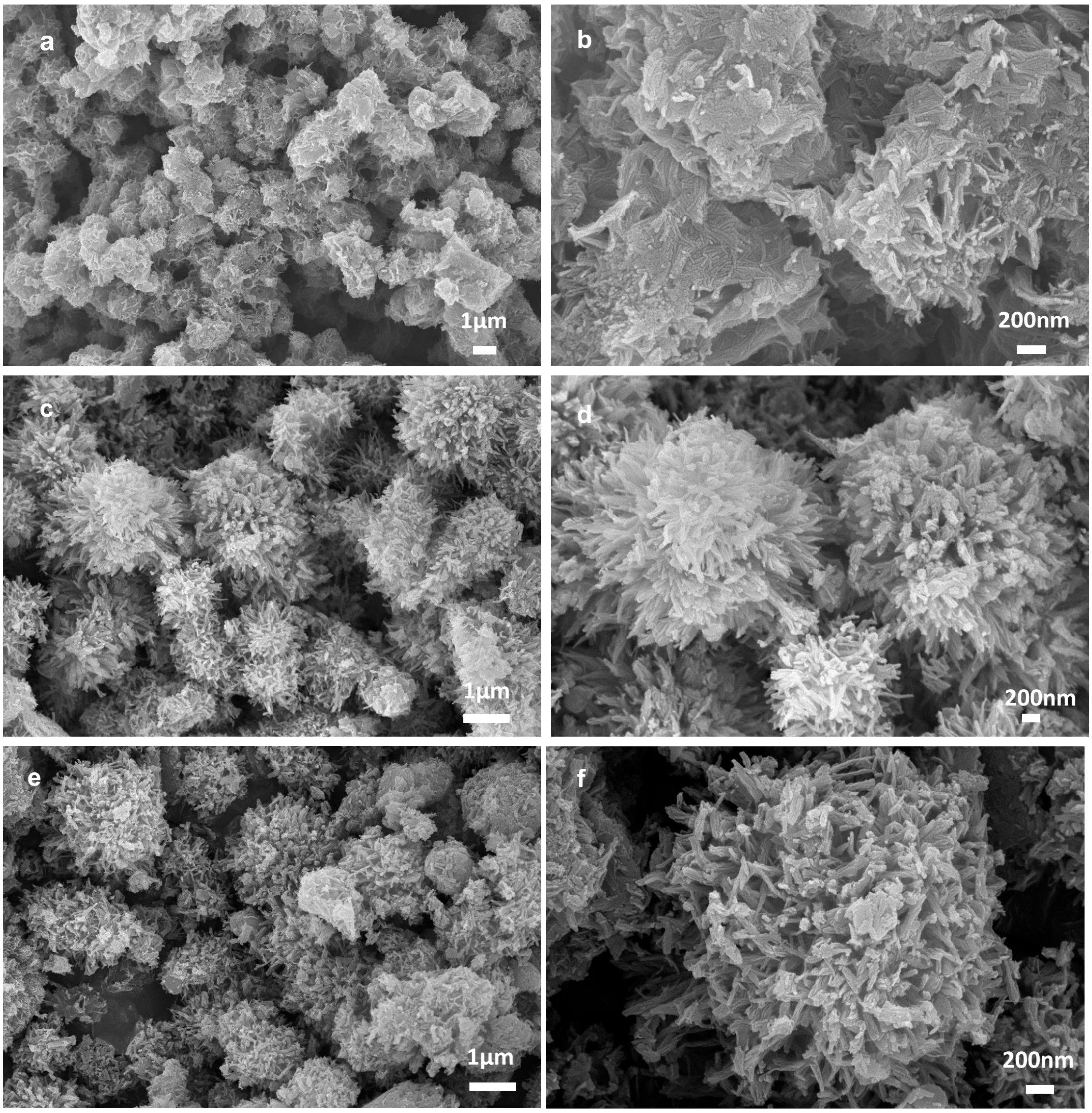


**Figure S2.** SEM images of Na-TNT (a, b), Na-UTMS (c, d) and H-UTMS (e, f).

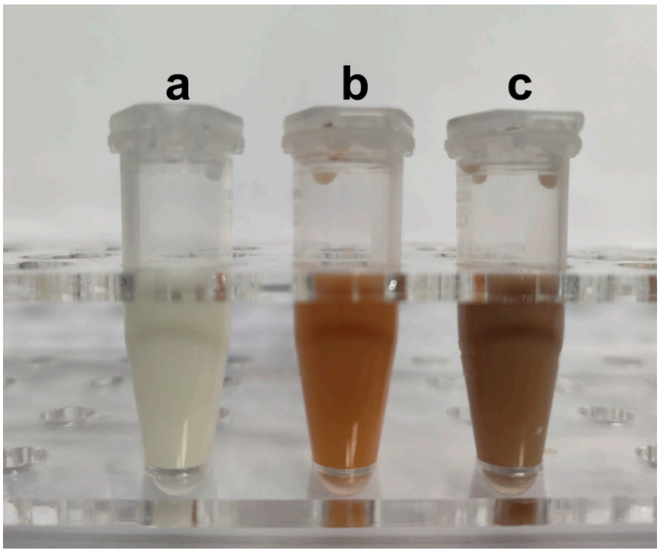


**Figure S3.** Photographs of H-UTMS (a), PDA-UTMS(b) and Cu-PDA-UTMS(c).

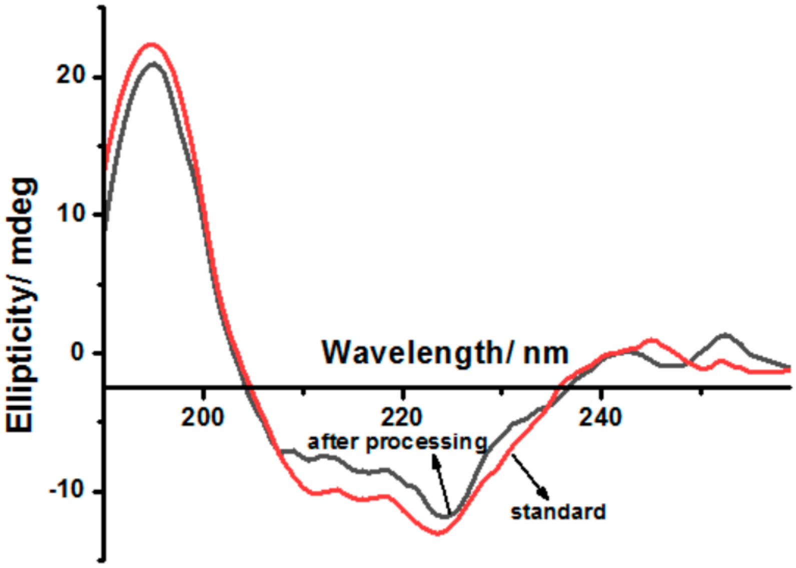


**Figure S4.** The circular dichroism (CD) of standard hemoglobin and hemoglobin solution after adsorption by the Cu-PDA-UTMS and recovery in 0.1% CTAB solution (The final hemoglobin solution is required to remove the CTAB molecule through dialysis).

**Table S1.** Elements content of H-UTMS, PDA-UTMS and Cu-PDA-UTMS by SEM/EDS

| Element | H-UTMS(Wt%) | PDA-UTMS(Wt%) | Cu-PDA-UTMS(Wt%) |
|---|---|---|---|
| Ti | 35.04 | 19.98 | 11.70 |
| C | - | 31.83 | 41.53 |
| N | - | 0.00 | 0.00 |
| O | 64.96 | 48.19 | 45.26 |
| Cu | - | - | 1.51 |
| Total | 100.00 | 100.00 | 100.00 |
